# Supplementary material for: Impact of ‘HIV-related stigma-reduction workshops’ on knowledge and attitude of healthcare providers and students in Central India: a pre-test and post-test intervention study
Source: BMJ Open. 2020 Apr 12;10(4):e033612. doi: 10.1136/bmjopen-2019-033612 (PMC7201299; doi:10.1136/bmjopen-2019-033612)
Supplement: Author's manuscript [file bmjopen-2019-033612.draft_revisions.pdf]

BMJ Open

BMJ Open is committed to open peer review. As part of this commitment we make the peer review history of every article we publish publicly available.

When an article is published we post the peer reviewers' comments and the authors' responses online. We also post the versions of the paper that were used during peer review. These are the versions that the peer review comments apply to.

The versions of the paper that follow are the versions that were submitted during the peer review process. They are not the versions of record or the final published versions. They should not be cited or distributed as the published version of this manuscript.

BMJ Open is an open access journal and the full, final, typeset and author-corrected version of record of the manuscript is available on our site with no access controls, subscription charges or pay-per-view fees (http://bmjopen.bmj.com).

If you have any questions on BMJ Open's open peer review process please email info.bmjopen@bmj.com

BMJ Open

Impact of 'HIV-related stigma-reduction workshops' on knowledge and attitude of healthcare providers and students in Central India: A pre- and post-test interventional study

Journal:	*BMJ Open*
Manuscript ID	bmjopen-2019-033612
Article Type:	Research
Date Submitted by the Author:	13-Aug-2019

Complete List of Authors:	Machowska, Anna; Karolinska Institute, Department of Public Health Sciences<br>Bamboria, Babu Lal; RD Gardi Medical College, Department of Medicine<br>Bercan, Courtney; Karolinska Institutet, Department of Public Health Sciences<br>Sharma, Megha; Karolinska Institutet, Department of Public Health Sciences; RD Gardi Medical College, Department of Pharmacology

Keywords:	People living with HIV/AIDS, Stigma and discrimination, Students of healthcare courses, Health Care Professionals, HIV-related stigma-reduction workshop

SCHOLARONE™
Manuscripts

I, the Submitting Author has the right to grant and does grant on behalf of all authors of the Work (as defined in the below author licence), an exclusive licence and/or a non-exclusive licence for contributions from authors who are: i) UK Crown employees; ii) where BMJ has agreed a CC-BY licence shall apply, and/or iii) in accordance with the terms applicable for US Federal Government officers or employees acting as part of their official duties; on a worldwide, perpetual, irrevocable, royalty-free basis to BMJ Publishing Group Ltd ("BMJ") its licensees and where the relevant Journal is co-owned by BMJ to the co-owners of the Journal, to publish the Work in this journal and any other BMJ products and to exploit all rights, as set out in our licence.

The Submitting Author accepts and understands that any supply made under these terms is made by BMJ to the Submitting Author unless you are acting as an employee on behalf of your employer or a postgraduate student of an affiliated institution which is paying any applicable article publishing charge ("APC") for Open Access articles. Where the Submitting Author wishes to make the Work available on an Open Access basis (and intends to pay the relevant APC), the terms of reuse of such Open Access shall be governed by a Creative Commons licence – details of these licences and which Creative Commons licence will apply to this Work are set out in our licence referred to above.

Other than as permitted in any relevant BMJ Author's Self Archiving Policies, I confirm this Work has not been accepted for publication elsewhere, is not being considered for publication elsewhere and does not duplicate material already published. I confirm all authors consent to publication of this Work and authorise the granting of this licence.

For peer review only

Impact of 'HIV-related stigma-reduction workshops' on knowledge and attitude of healthcare providers and students in Central India: A pre- and post-test interventional study

Anna Machowska[1#], BL Bamboria[2#], Courtney Bercan[1], Megha Sharma[1,3§]

[1] Department of Public Health Sciences, Karolinska Institutet, Stockholm, Sweden

[2] Department of Medicine, Ruxmaniben Deepchand Gardi Medical College, Ujjain, India

[3] Department of Pharmacology, Ruxmaniben Deepchand Gardi Medical College, Ujjain, India

[§]Corresponding author:

Dr. Megha Sharma

Researcher,

Department of Public Health Sciences, Karolinska Institutet, Stockholm, Sweden

Associate Professor,

Department of Pharmacology, R.D. Gardi Medical College, Surasa, MP, India

e-mail: megha.sharma@ki.se

[#] These authors have contributed equally to the work

E-mail addresses of authors:

Anna.machowska@ki.se

drblbamboria53@gmail.com

courtney_bercan@hotmail.com

megha.sharma@ki.se, meghasharma27@rediffmail.com

For peer review only

Abstract

Introduction: People living with human immunodeficiency virus (HIV) and acquired immunodeficiency syndrome (AIDS) experience stigma and discrimination throughout their life. The consequences of stigma and discrimination are severe when enacted by healthcare providers (HCPs) and result in delay or poor adherence to the treatment. Studies have demonstrated universal stigma amongst HCPs, yet only a few have presented the effect of interventions on the knowledge and attitude of practising- (doctors and nurses) and future-HCPs (students of healthcare courses).

Objectives: The primary objective was to evaluate knowledge, attitude and infection risk perceptions related to HIV/AIDS among practising- and future-HCPs in Central India. Secondly, the impact of the 'HIV-related stigma-reduction workshop' using a pre- and post-intervention study design was evaluated.

Settings and participants: The study was conducted during January-August 2016 at two tertiary-care hospitals and three colleges of healthcare courses in Ujjain district of Central India. Overall, 650 individuals; HCPs (clinicians-75, nurses-211), and students (medical-205, nursing-123 and Diploma in Medical and Laboratory Technology [DMLT]-36); voluntarily participated in the study.

Intervention: The interventional workshop contained in 3 sessions and included training in epidemiology, transmission and treatment of HIV infection, rights of infected persons, duties of HCPs, and use of standard precautions.

Results: At pre-intervention, DMLT students had the lowest and clinicians the highest knowledge scores (24% vs 45%). The stigmatised attitude reflected in all groups, lowest among clinicians (21%) and highest among DMLT students (34%). Improvement in the post-intervention

For peer review only

For peer review only - http://bmjopen.bmj.com/site/about/guidelines.xhtml

knowledge scores was highest in medical students (36%) and least among clinicians (16%). The participants' attitude improved between 3% to17% in all groups.

Conclusions: Significant post-intervention improvements were seen in both knowledge and attitude in all groups. Students had a higher tendency to improve than the HCPs. Further studies are needed to conclude that this specific intervention can change long –term behaviour of HCPs.

Article Summary

Strengths and limitations of this study

- The study demonstrate a positive effect of the 'HIV-related stigma-reduction workshop' educational intervention on knowledge, attitude, and discrimination of HCPs and students of healthcare courses, towards PLWHA.
- The intervention used low-resources, and therefore could be adopted and scaled up to train HCPs in other similar settings.
- As the study was conducted at work places or educational institutes, the responses are subjected to a social-desirability bias, and thus might result in underestimation of stigma and discrimination among the participants.
- The end-goal of any stigma-reduction intervention is to reduce stigmatizing behaviours, but as these behaviours are difficult and complex to capture and quantify at the workplace, we were restricted to capture attitudes as a proxy.
- The post-intervention questionnaire was filled in immediately following the intervention. Thus, the long-term effect cannot be claimed.

For peer review only

Introduction

The human immunodeficiency virus (HIV) and acquired immunodeficiency syndrome (AIDS) epidemic is a global challenge. As per an estimation made at the end of 2017, 36.9 million people were either infected by HIV or were living with AIDS, globally. Of these, about 22 million people could access antiretroviral therapy (ART), and 940 000 people died from HIV-related illnesses (1, 2). The United Nations' 90-90-90 targets launched in 2014 have become a central pillar of global efforts to end the HIV/AIDS epidemic. The aim is to diagnose 90% of all HIV-positive persons, provide ART for 90% of those diagnosed, and achieve viral suppression for 90% of those treated by 2020. The program estimates to result in 73% of people with HIV achieving viral suppression, a crucial step in ending the AIDS epidemic by 2030 (3). One key aspect of the HIV/AIDS epidemic that is significantly affected by culture and knowledge of HIV transmission is stigma, specifically HIV/AIDS-related stigma. HIV stigma can be described as a socially shared perception of the devalued status of PLWHA (4). The presence of stigma and discrimination against PLWHA at various societal levels fuels the HIV-epidemic, and the consequences are more severe if present among the healthcare providers (HCPs).

HCPs are the core and integral part of the healthcare delivery system. HCPs play a pivotal role in creating a positive environment for better health outcomes of PLWHA and preventing viral transmission (5). However, inadequate knowledge about the HIV infection epidemiology among HCPs gives rise to fear and misconceptions about transmission of the infection (6-8). Fear and misconceptions alter the attitude and enhance the development of stigma and discrimination towards PLWHA (9). Some of the major consequences are, delay in seeking healthcare, impaired timely diagnosis, unwillingness to disclose the HIV-status in the community and while seeking healthcare, and delay or non-compliance to the ART (5, 6). Undisclosed HIV status puts the society

For peer review only

For peer review only - http://bmjopen.bmj.com/site/about/guidelines.xhtml

at higher risk of infection transmission, contributes to increased morbidity and mortality of PLWHA, increased healthcare costs and burden on the overall healthcare sector (10-12). Therefore, in addition to diagnosis and treatment, provision of discrimination- and stigma-free healthcare is a prerequisite to achieve the sustainable development target to end the epidemic.

In 2016, with the prevalence of 2.1 million PLWHA, India ranked 3rd in the world, after South Africa and Nigeria (13, 14). The prevalence of PLWHA varies state-wise the country. However, the threat of the spread of the infection is high throughout the country due to high inter-state and inter-district migrations (15). Global organisations and governments of high infection risk countries, including Indian, have prioritised stigma-reduction through various interventional programs (16). In India, the National AIDS Control Organization (NACO) advocates the prohibition of social discrimination against PLWHA (14, 16). NACO has identified seven districts of the state as high HIV-prevalence districts. Ujjain division is an administrative unit which includes four of these high-risk districts, i.e. Neemuech, Mandsour, Ujjain, and Ratlam (17, 18). However, the available data from various parts of the country shows that PLWHAs have experienced stigma and discrimination from the community and the HCPs (19, 20).

Educational interventions intended to improve knowledge about HIV/AIDS are effective to reduce the stigma among HCPs and improved healthcare delivery (21-26). The stigma-reduction intervention studies, in general, are time-consuming and resource-intensive. Most of the interventional studies had mainly focused on nurses and nursing students and were not focused on clinicians and students of the healthcare courses (11, 23-25). The students of healthcare courses such as medical, laboratory technician and nursing courses, are future HCPs. It is, therefore, important to develop appropriate interventions to improve the knowledge and reduce HIV-related stigma, targeting both the practising (clinicians and nurses) and future (the students) HCPs (27).

For peer review only - http://bmjopen.bmj.com/site/about/guidelines.xhtml

However, little is known about HIV/AIDS-related knowledge, attitudes and perceived infection risk of the practising and future HCPs from India.

Objectives

To evaluate the knowledge and attitude and infection risk perceptions related to HIV/AIDS, among the HCPs, and the students of healthcare courses, i.e. medical, laboratory technician and nursing in Ujjain district in Central India. Further, to assess and compare the impact of the 'HIV-related stigma-reduction workshop' intervention on the participant groups using a pre-and post-intervention study design.

Methods

This interventional study was conducted between January 2016 and August 2016 at three colleges of healthcare courses and two tertiary-care hospitals in Ujjain district in Madhya Pradesh state in India. One of the hospitals is located in a rural area and is associated with all three colleges included in the study. One of the two study hospitals is associated with a medical college, a nursing college and a DMLT college. This hospital is referred as teaching hospital (TH), and the students of Bachelor of Medicine, Bachelor of Surgery (MBBS), are referred as medical students in the present communication. An ART centre is located at the TH with specialized healthcare facilities for the PLWHA. Another participating hospital is located in Ujjain city, referred to as a non-teaching hospital (NTH).

Participants

For peer review only - http://bmjopen.bmj.com/site/about/guidelines.xhtml

The participants were practicing HCPs clinicians and nurses, and future HCPs, i.e. students of medical, nursing and Diploma in Medical and Laboratory Technology [DMLT] courses, enrolled in respective institutes in Ujjain district of Central India.

Sampling strategy

All groups of the HCPs and students were invited to participate in the workshop through various means such as leaflets posted at the notice boards, announcements during lectures or meetings. The leaflets were written in Hindi and English to offer equal chances for all potential participants to learn about and attend the workshop to enhance their HIV/AIDS-related knowledge and ways to stay protected at the workplace. The workshops were conducted at respective colleges and hospitals for providing ease to participate.

Inclusion and exclusion criteria

Participation was voluntary, and the participant could leave the session at any time without experiencing any repercussions. Participants who could not attend or complete both pre- and post-workshop sessions were not included in the analysis (Figure 1).

Figure 1 Inclusion and exclusion criteria of the study participants (make in 1,200 dpi)

Intervention

Structure of the workshops

All workshops were conducted by the same facilitators: a clinician and an officer-in-charge of the ART centre at the TH and the leader of hospital infection prevention and control team at the TH and the NTH. In total, 13 workshops were conducted. Participants were divided into smaller

For peer review only - http://bmjopen.bmj.com/site/about/guidelines.xhtml

For peer review only

subgroups as follows: clinicians (2 groups), nurses (5 groups), medical students (3 groups), nursing students (2 groups) and DMLT students (1 group) to ensure that they feel comfortable in the learning environment.

Content of the workshops

The workshop was 2 hours long and was divided into three sessions: the 1st session consisted of an animated video demonstrating the HIV infection epidemiology, mechanism of viral transmission, diagnostic testing, treatment options, mechanism of action of ART drugs and ethical issues related to PLWHA and ART. This was followed by 2nd session with a powerpoint presentation focusing the topics such as rights of PLWHA, duties of HCPs and predictable consequences due to stigma and discrimination against PLWHAs. The 3rd session was more interactive and dynamic. In this part, information about universal precautions and preventive measures for infection prevention and control was provided, describing the importance of the use of standard precautions, including hand hygiene in daily routine. It was explained how and when to use gowns, gloves, face shields, masks and goggles, availability of post-exposure prophylaxis (PEP) in the study settings and use of in-house prepared alcohol-based hand rub, i.e. 'Micro *Kavach*' (28). This was followed by the demonstration and practice of the WHO recommended hand hygiene techniques using 'Micro *Kavach*' (28). A time period of 15 to 20 minutes was assigned for open discussion that was followed by filling-up the post-intervention questionnaires.

Data collection and analysis

The context-specific pre- and post-intervention questionnaires were developed locally and used as data collection tools. The questionnaires were pre-tested in each potential participant groups and modified based on the received feedback. Questionnaires were formatted in both English and

For peer review only

For peer review only - http://bmjopen.bmj.com/site/about/guidelines.xhtml

175 Hindi, divided into following sections: (1) participants' demographic information, (2) experiences, (3) HIV/AIDS-related knowledge, (4) attitudes towards PLWHA, (5) risk perception and willingness to provide care to PLWHA and patients with Hepatitis B.

The participants were asked to fill in the questionnaire once before the workshop (pre-intervention) and once 20 minutes after the workshop (post-intervention).

180 The authors scored the responses related to the knowledge and attitude questions. Each correct response in the knowledge section received a score of one, and each incorrect response received a score of zero. Scores of each individual were added and converted to the percentage of correct responses pre and post-intervention. Based on the previous research in this field levels of knowledge were categorised into "low" for participants who scored 50% and below, "moderate" for those who scored between 51% and 74%, and "high" for those who scored 75% and above (29).

The responses to the questions related to the participants' attitude towards PLWHA were classified into three categories: positive (non-stigmatizing), unsure and negative (30). A positive response was given a score of one and the responses where the participants were unsure or, where the responses indicated negative attitude including fear, stigma, or intent to discriminate PLWHA, were given a score of zero (31). Scores of the participants were added and converted to the percentage of correct responses pre- and post-intervention. Question for which the missing data was higher than 50% were excluded from the analysis.

Risk perception questions were categorical and dichotomised according to the outcome of interest for example (a) I am willing or not willing to care for a patient, or (b) I am at high risk or not at high risk of HIV exposure at the workplace.

For peer review only - http://bmjopen.bmj.com/site/about/guidelines.xhtml

Statistical analysis

The data on demographic and basic HIV-related information is presented as mean and standard deviations for continuous variables and as percentages and frequencies for categorical variables. The knowledge, attitudes and work environment variables are presented as median with interquartile range (IQR). The difference between the pre- and post-intervention scores were analysed using a Wilcoxon signed-rank test for continuous variables and McNemar's test for categorical variables. The score difference was calculated by subtracting the pre-intervention (baseline) score from the post-intervention score and represents the percentage unit increase or decrease in relation to the baseline score. As these scores were not normally distributed, a Kruskal-Wallis test was conducted to determine a statistically significant difference among the participant groups in pre-intervention and post-intervention scores as well as the difference of scores. Post-hoc Dunn's tests were conducted to evaluate the relationship between the groups. The statistical significance was set at the level of $p<0.05$. Data collected in the questionnaires were entered using Epidata software (version 3.1) and Microsoft Excel. Statistical analysis was performed using the Statistical Package for the Social Sciences (SPSS and STATA) software.

Results

Baseline characteristics

A total of 920 invited persons, 746 participants (81%) voluntarily participated in the study. Out of 746 participants, 650 attended all intervention sessions and filled-in both pre- and post-intervention questionnaires. Of 650 participants, 56% were students, and 44% were practising HCPs. Thus, the data of 650 participants were included in the analysis (Figure 1).

Figure 2 Inclusion and exclusion criteria of the study participants

For peer review only - http://bmjopen.bmj.com/site/about/guidelines.xhtml

The mean age of the participants was 23 years, the majority were of the Hindu faith, and 56% were males (Table 1). The proportion of participants who had received HIV/AIDS-related training previously, varied from 4% in the clinician group to 78% in the DMLT student group. Only 2% of medical students stated that they had worked with PLWHA before. A high proportion of participants in each group reported an experience of accidental exposure to HIV or PLWHA. A range of 9% to 20% of the participants admitted discriminating against PLWHA at workplaces. Six (3%) of medical students and up to 11% of DMLT students admitted that they had refused to provide care for PLWHA during their scheduled clinical visits. A range of 17% to 28% of participants thought that their colleagues discriminate against PLWHA (Table 1).

Table 1 Baseline demographic information (Part A) and experiences (Part B) of the participants

	Clinicians (n=75)	Nurses (n=211)	Medical students (n=205)	Nursing students (n=123)	DMLT students (n=36)
**Part A: Baseline demographic information**					
**Age, mean (SD)**	28 (4)	25 (6)	20 (1)	21 (3)	20 (2)
**Sex, n (%)**					
Male	30 (40)	148 (71)	109 (53)	51 (42)	28 (78)
Female	45 (60)	63 (30)	96 (47)	72 (59)	8 (22)
**Married, n (%)**	21 (28)	70 (33)	-	11 (9)	35 (97)
**Religion, n (%)**					
Hindu	62 (83)	192 (91)	183 (89)	120 (98)	31
Muslim	2 (3)	5 (2)	6 (3)	3 (2.4)	4
Christian	2 (3)	10 (5)	1 (0.5)	-	-
Other	9 (12)	4 (2)	15 (7)	-	2
**Have you ever attended any HIV/AIDS related training?**	3 (4)	39 (18)	63 (31)	28 (23)	28 (78)
**Part B: Experiences of participants**					
**Have you ever cared for PLWHA before? n (%)**	37 (49)	121 (57)	4 (2)	88 (72)	18 (50)
If yes, how many times?					
< 10	29 (78)	59 (49)	2 (50)	61 (69)	5 (28)
< 50	2 (5)	27 (22)	-	14 (20)	8 (44)
> 100	1 (3)	19 (16)	-	-	5 (28)
Cannot recall the numbers	-	16 (13)	2 (50)	13 (15)	-
**§Have you ever had an accidental exposure with body fluid of PLWHA at work?**	§4 (11)	§24 (20)	§3 (75)	§14 (16)	§1 (6)
**Have you ever refused to care for PLWHA?**	6 (8)	18 (9)	6 (3)	6 (5)	4 (11)
**Have you discriminated against PLWHA at the workplace?**	7 (9)	42 (20)	22 (11)	13 (11)	5 (19)

For peer review only - http://bmjopen.bmj.com/site/about/guidelines.xhtml

**Do you think your colleagues discriminate against PLWHA?**	21 (28)	45 (21)	51 (25)	28 (23)	6 (17)
**Is your family aware that you ought to care for PLWHA patients as well**	49 (65)	134 (64)	127 (62)	100 (81)	16 (72)
**Is there any family pressure regarding caring for PLWHAs**	15 (20)	46 (22)	16 (8)	15 (12)	8 (22)
**Do you have any friend or family who is identified as PLWHA?**	3 (4)	16 (8)	3 (2)	5 (4)	2 (6)
**There is enough equipment to maintain universal precautions at work place.**	20 (27)	126 (60)	79 (39)	84 (68)	19 (53)
**Have you ever had any accidental exposure to HIV through PLWHA at work?**	4 (5)	24 (11)	3 (1)	14 (11)	1 (3)

DMLT= Students of Diploma in Medical and Laboratory Technology; Medical students= Students of Bachelor of Medicine, Bachelor of Surgery; PLWHA= people living with HIV/AIDS

The boxes where totals do not add up to 100%, the remaining percentage of the participants did not respond to that question. §Proportion calculated from patients who confirmed that they have ever cared for PLWHA

Impact of the intervention on knowledge scores

Pre-intervention knowledge score, presented as the percentage of correct responses, showed the low overall level of knowledge with the poorest in DMLT students (22%) and the highest among the clinicians (45%, Table 2). Post-intervention knowledge scores ranged from 48% in the nurses' group to 64% in the medical students' group. The post-intervention knowledge scores were significantly higher than pre-intervention knowledge scores (p <0.001, Table 2), and almost all groups reached a moderate level of knowledge. The difference between knowledge scores of all study groups was statistically significant (p<0.001). The knowledge scores of the students of both medical and nursing courses improved more than their respective practising HCPs. Pairwise comparison showed that at the pre-intervention stage, the clinicians had the highest knowledge than the rest of the groups (p<0.001, Table 3). However, in the post-intervention stage, the medical students presented higher knowledge than the clinicians while the knowledge of nurses was lowest in the sequence.

Impact of the intervention on attitude

Evidence of stigmatised attitude was observed among all responder groups. The lowest level of stigmatising attitudes at pre-intervention was found in the clinicians' group, where 79% responded

For peer review only - http://bmjopen.bmj.com/site/about/guidelines.xhtml

to the questions without stigma. On the other hand, in the DMLT students' group, only 66% of responses presented a non-stigmatising attitude (Table 2). In post-intervention, the percentages of non-stigmatising responses were higher, ranging from 79% (lowest) among the nurses to 84% in the medical students' group. The attitude improved significantly across all groups, with 3% in the clinician group and 17% in the DMLT students' group (p<0.001). Overall, the intervention resulted in an improvement in stigma levels to 21%, as shown in the nurses' group. Pairwise comparison showed that medical students had the highest improvement in their attitude compared to the nurses, nursing students, as well as the clinicians (Table 4).

Table 2. Pre- and post-intervention scores of knowledge and attitude among the participant groups

**Knowledge**					
	**Clinicians (n=75) % (IQR)**	**Nurses (n=211) % (IQR)**	**Medical students (n=205) % (IQR)**	**Nursing students (n=123) % (IQR)**	**DMLT students (n=36) % (IQR)**
**Percentage of correct responses**					
**Pre-Intervention,** median (IQR)	45 (15)	30 (19)	29 (14)	30 (19)	22 (14)
**Post-Intervention,** median (IQR)	59 (14)	48 (26)	64 (21)	59 (26)	52 (17)
**Difference**	14*	18*	35*	29*	30*
**Attitude**					
**Percentage of responses without stigma**					
**Pre-Intervention,** median (IQR)	79 (18)	74 (15)	74 (18)	76 (12)	66 (35)
**Post-Intervention,** median (IQR)	82 (11)	79 (12)	84 (16)	82 (9)	83 (14)
**Difference**	3*	5*	10*	5*	17*

*Statistically significant differences with significance level p< 0.05.
DMLT= Students of Diploma in Medical and Laboratory Technology; IQR= Interquartile range; Medical students= Students of Bachelor of Medicine, Bachelor of Surgery

Table 3. Pre- and post-intervention pairwise comparison of knowledge between the participant groups

**Groups Compared**	**Pre-Intervention Adjusted significance**	**Post-Intervention Adjusted significance**	**Pre- and Post-Intervention Adjusted significance**

For peer review only - http://bmjopen.bmj.com/site/about/guidelines.xhtml

DMLT students- Nursing students	<0.05*	0.068	1.000
DMLT students - Medical students	<0.01*	<0.001*	0.629
DMLT students -Clinicians	<0.001*	0.116	<0.001*
DMLT students - Nurses	0.059	1.000	0.234
Medical students - Clinicians	<0.001*	0.051	<0.001*
Nursing students - Clinicians	<0.001*	1.000	<0.001*
Nursing students – Medical students	1.000	<0.05*	0.934
Nurses- Nursing students	1.000	<0.001*	<0.001*
Nurses- Clinicians	<0.001*	<0.001*	<0.05*
Nurses– Medical students	1.000	<0.001*	<0.001*

*Statistically significant differences with significance level $p < 0.05$.
Significance values have been adjusted by the Bonferroni correction for multiple tests. DMLT= Students of Diploma in Medical and Laboratory Technology; Medical students= Students of Bachelor of Medicine, Bachelor of Surgery

Table 4. Pre- and post-intervention pairwise comparison of attitudes between the participant groups

**Groups Compared**	**Pre-Intervention Adjusted significance**	**Post-Intervention Adjusted significance**	**Pre- and Post-Intervention Adjusted significance**
DMLT students - Nursing students	0.184	1.000	0.147
DMLT students - Medical students	0.479	0.614	1.000
DMLT students -Clinicians	<0.01*	1.000	0.134
DMLT students - Nurses	0.627	1.000	0.439
Medical students - Clinicians	<0.05*	1.000	<0.01*
Nursing students - Clinicians	0.252	1.000	1.000
Nursing students – Medical students	1.000	0.173	<0.01*
Nurses- Nursing students	1.000	0.614	1.000
Nurses- Clinicians	<0.05*	0.092	1.000
Nurses– Medical students	1.000	<0.001*	<0.05*

*Statistically significant differences with significance level $p < 0.05$. Significance values have been adjusted by the Bonferroni correction for multiple tests.
DMLT= Students of Diploma in Medical and Laboratory Technology; Medical students= Students of Bachelor of Medicine, Bachelor of Surgery

Table 5. Risk perception reported by the participants in pre- and post-intervention questionnaires: Proportion of the participants who responded as “Yes” to the questions asked

For peer review only

For peer review only - http://bmjopen.bmj.com/site/about/guidelines.xhtml

Questions	Time point	Nurses (n=211) n (%)	Clinicians (n=75) n (%)	Nursing students (n=123) n (%)	Medical students (n=205) n (%)	DMLT students (n=36) n (%)
Do you think you are at high risk for HIV/AIDS at work?	Pre-intervention	71 (34)	50 (67)	39 (32)	61 (30)	22 (61)
	Post-intervention	81 (38)	51 (68)	86 (70)*	82 (40)*	17(47)
Are you willing to care for-	Pre-intervention	174 (83)	64 (85)	112 (91)	155 (76)	28 (78)
a) Patients with hepatitis B	Post-intervention	192 (91)*	65(87)	118 (96)	196 (96)*	32 (89)
b) PLWHA§	Pre-intervention	151 (72)	65 (87)	114 (93)	179 (87)	34 (94)
	Post-intervention	175 (83)*	66 (88)	110 (89)	183 (89)	30 (83)

*Statistically significant differences with significance level $p< 0.05$. DMLT= Students of Diploma in Medical and Laboratory Technology; Medical students= Students of Bachelor of Medicine, Bachelor of Surgery; PLWHA= People living with HIV/AIDS. [§]The question was also classified as an attitude question.

Impact of the intervention on infection risk perception

Thirty per cent of medical students and 67% of the clinicians perceived themselves to be at high risk of HIV infection in their workplace at the pre-intervention stage. Willingness to care for PLWHA was relatively high at the pre-intervention, ranging from 72% among the nurses to 94% among the DMLT student group. The proportion of participants who were willing to care for people living with Hepatitis B was similar to those willing to care for PLWHA.

After the workshop, the medical and nursing student reported a significant increase in awareness of risk perception of HIV infection compared to their statements at pre-intervention. There was a significant increase in post-intervention willingness to care for PLWHA in the nursing group, and a non-significant decrease in the DMLT and nursing students' group (Table 5).

Discussion

The present study evaluates the effect of 'HIV-related stigma-reduction workshops' and showed that there was a significant improvement in HIV/AIDS-related knowledge in all groups who participated in the intervention irrespective of their inadequate baseline knowledge. Our study highlights that stigmatising attitudes are widespread among practising HCPs and students of

For peer review only

For peer review only - http://bmjopen.bmj.com/site/about/guidelines.xhtml

various healthcare courses. However, this stigmatising attitude and discrimination to provide medical care to PLWHA reduced significantly among the participants after the intervention. The student groups showed more receptiveness to improve their knowledge and attitudes towards PLWHA than the practising HCPs.

Knowledge

Overall, the study participants had inadequate HIV/AIDS-related knowledge, and the clinicians had the highest pre-intervention knowledge score among all groups. The low level of knowledge presented in our study is comparable with results from a cross-sectional survey conducted among consultants, residents, medical students, laboratory technicians, and nurses, in Uttarakhand, India (32). In this study, the clinicians had better knowledge than the rest of the groups, while the overall knowledge was not complete.

The implication of acquired knowledge and attitudes are complex, but theories suggest that knowledge is associated with baseline beliefs and attitudes and depends on the readiness of the learners and their willingness to integrate the change into their behaviour (33, 34). Our study showed the lowest increase in post-intervention knowledge scores among clinicians and the highest increase among the student groups. This could be due to several factors. Firstly, clinicians showed adequate knowledge at pre-intervention, thus had less scope to improve their knowledge at post-intervention. Secondly, as per the educational theory; the recognition of the gap in knowledge, skills, or the need for behaviour change is essential for the desired change (33, 34). The student groups, being in the learning phase of life, may have been more receptive to the training from the beginning and, thus improved more than the rest of the groups (33, 34).

For peer review only - http://bmjopen.bmj.com/site/about/guidelines.xhtml

In our study, the overall knowledge scores increased substantially after intervention in all groups ranging, from 14% in clinicians to 35% in the medical students' group. Two single centre studies conducted among nursing students in Bengaluru and nurses in Delhi, India evaluated the effect of a brief stigma-reduction curriculum and educational intervention on HIV knowledge (23, 25). In Bengaluru, the intervention was divided into two sessions of 1-hour each while in Delhi; it was a 2-days training program. The educational intervention delivered showed significant improvement in knowledge scores in both settings. An increase of 9% post-intervention knowledge in Bengaluru and 22% in Delhi was presented.

Although, in both studies, the participants attended two educational sessions, contrary to our single workshop; the improvement in our study was higher. Therefore, it could be inferred that the number and duration of the educational workshops do not correspond to improve the knowledge.

Attitude

The percentage of non-stigmatizing responses ranged from 66% in the DMLT students to 79% among the clinicians. Although the presence of stigma and discrimination was visible in the pre-intervention attitude scores across all participant groups, the results are comparable to the previously mentioned cross-sectional study by *Doda et al.* in Uttarakhand, India where the median of positive responses ranged from 69% in postgraduate residents to 92% in consultants (32).

Professional experience in working with PLWHA is a predictor of more positive attitudes towards PLWHA (14,27). The higher levels of stigmatising attitudes found among participants (students) in our study could be due to incomplete or inadequate knowledge, and also relatively less experience of working with PLWHA.

For peer review only
For peer review only - http://bmjopen.bmj.com/site/about/guidelines.xhtml

One of the primary outcomes of our workshops was the reduction of negative attitudes ranging from 3% in clinicians to 17% in DMLT students. Our results are comparable with a study from China among clinicians, nurses, and laboratory technicians (35) which showed 13% and 22% reduction in stigmatising attitudes at 6 and 12 months respectively after the intervention. On the other hand, the reduction presented in our study is lower than the average reduction shown in a study from Pune, India (33%). The Pune based study examined the impact of a 4-day long HIV/AIDS health education program on the knowledge and positive attitudes toward caring for HIV-infected patients of nurses (24).

Risk perception

Another highlight of our study was willingness shown by the majority of HCPs and students, to care for PLWHA, ranging from 72% to 94%, at the pre-intervention stage. Our findings are in line with a study conducted in India by *Kermode et al.* showing a relatively high willingness of HCPs to care for PLWHA in spite of the presence of the stigmatising attitudes (7). However, our results differ from the studies conducted in Russia, showing that only 20% of HCPs were willing to care for PLWHA (20%) (36).

Medical and nursing students significantly improved their awareness of being at risk for HIV/AIDS at work. The willingness of the participants to change behaviour might have influenced this improvement. There is a lack of studies that had assessed the pre- and post-intervention risk perception among HCPs. There was an interplay between the knowledge of risks and universal precautions in our study, actual risk (due to the prevalence of HIV and job description), the environmental barriers and lack of accessibility of protective equipment. This interplay point towards a need for structural changes at the workplace in conjunction with customised educational

For peer review only - http://bmjopen.bmj.com/site/about/guidelines.xhtml

interventions to reduce risk perception (6, 7). Further studies are needed to determine the factors affecting willingness among HCPs to care for PLWHA.

Conclusions

Combating stigma is a key priority to achieve success in epidemic control and providing quality care to PLWHA. The findings from this study show that the ´HIV-related stigma-reduction workshops'- a brief and relatively low resource intervention successfully increased knowledge and reduced stigma in both HCPs and students.

We, therefore, recommend repetitive conduction of such contextualized workshops at healthcare facilities. Attention must be drawn to interventions focusing on stigma-reduction that can be scaled up and transferred to multiple HCPs groups, including both practicing- and future-HCPs. We also recommend to conduct similar contextualized workshops in other settings to explore sustainability and evaluate the impact in those settings. Incorporate more practical issues effectively in the teaching curricula of all healthcare courses, is of high importance. Moreover, the inclusion of the qualitative component, the use of extended follow-up and adding behavioural assessment in future studies is recommended to understand participants' attitudes and willingness to change the stigmatising behaviour for PLWHA.

Acknowledgements

We thank all the study participants, the management of the hospitals and colleges for facilitating and supporting the study. We are thankful to Mr Amit Pawar for assisting in data collection and data management.

Footnotes

For peer review only - http://bmjopen.bmj.com/site/about/guidelines.xhtml

Author contributions

MS and BLB perceived the study. MS and BLB designed and performed the questionnaire and the content of the interventions, and collected the pre-and post-intervention data. AM, CB and MS performed the analysis. AM and MS drafted the article. AM, MS, BLB and CB gave critical comments to modify the manuscript. All authors gave final approval to the present version for publication.

Funding

This work was supported by Swedish Research Council grant number 2017-01327.

Competing interests

Authors have no conflicts of interests to declare.

Ethical considerations

Ethical approval was granted by the local ethics committee (311/17-07-2013 and 249/23-01-2013). Verbal and written consents were obtained from each participant before the study. Participants were assured by the researchers for full confidentiality. Participants were encouraged to respond to the questionnaires fearlessly and were ensured for no repercussions based on the responses.

A data sharing statement

The data are not publicly available at present because it contains information that could compromise the confidentiality or consent of the study participants.

For peer review only

For peer review only - http://bmjopen.bmj.com/site/about/guidelines.xhtml

Patient consent for publication

Not required.

For peer review only
For peer review only - http://bmjopen.bmj.com/site/about/guidelines.xhtml

References

1. Organization WH. Sustainable Development Goals 2015 [Available from: http://www.un.org/sustainabledevelopment/sustainable-development-goals/.
2. Organization TWH. Global Health Observatory (GHO) data: HIV/AIDS 2019 [Available from: https://www.who.int/gho/hiv/en/.
3. UNAIDS. 90–90–90—An ambitious treatment target to help end the AIDS epidemic 2017 [Available from: https://www.unaids.org/en/resources/documents/2017/90-90-90.
4. HIV/AIDS JUNPo. Stigma and discrimination 2003 [Available from: http://data.unaids.org/Publications/fact-sheets03/fs_stigma_discrimination_en.pdf.
5. Chambers LA, Rueda S, Baker DN, Wilson MG, Deutsch R, Raeifar E, et al. Stigma, HIV and health: a qualitative synthesis. BMC Public Health. 2015;15:848.
6. Datta S, Bhattacherjee S, Sherpa PL, Banik S. Perceived HIV Related Stigma among Patients Attending ART Center of a Tertiary Care Center in Rural West Bengal, India. J Clin Diagn Res. 2016;10(10):VC09-VC12.
7. Kermode M, Holmes W, Langkham B, Thomas MS, Gifford S. HIV-related knowledge, attitudes and risk perception amongst nurses, doctors and other healthcare workers in rural India. Indian J Med Res. 2005;122(3):258-64.
8. Vorasane S, Jimba M, Kikuchi K, Yasuoka J, Nanishi K, Durham J, et al. An investigation of stigmatizing attitudes towards people living with HIV/AIDS by doctors and nurses in Vientiane, Lao PDR. BMC Health Serv Res. 2017;17(1):125.
9. Link BG, Phelan JC. Conceptualizing Stigma. Annual Review of Sociology. 2001;27(1):363-85.
10. Steward WT, Bharat S, Ramakrishna J, Heylen E, Ekstrand ML. Stigma is associated with delays in seeking care among HIV-infected people in India. J Int Assoc Provid AIDS Care. 2013;12(2):103-9.
11. Ekstrand ML, Ramakrishna J, Bharat S, Heylen E. Prevalence and drivers of HIV stigma among health providers in urban India: implications for interventions. J Int AIDS Soc. 2013;16(3 Suppl 2):18717.
12. Katz IT, Ryu AE, Onuegbu AG, Psaros C, Weiser SD, Bangsberg DR, et al. Impact of HIV-related stigma on treatment adherence: systematic review and meta-synthesis. J Int AIDS Soc. 2013;16(3 Suppl 2):18640.
13. data U. 2017 [Available from: http://www.unaids.org/sites/default/files/media_asset/20170720_Data_book_2017_en.pdf.
14. Organization NAC. 2016-2017 [Available from: http://naco.gov.in/sites/default/files/NACO%20ANNUAL%20REPORT%202016-17.pdf.
15. WELFARE MPsAcsDOHF. [Available from: http://www.mpsacsb.org/MPSACS-introduction.php.
16. Bagcchi S. India passes law prohibiting discrimination against people with HIV/AIDS. BMJ. 2017;357:j1973.
17. , Office of the Registrar General & Census Commissioner I. Census of India 2011 [Available from: http://censusindia.gov.in/2011-prov-results/data_files/mp/01Content.pdf.
18. Economic and Human Development Indicators 2011 [Available from: http://www.undp.org/content/dam/india/docs/madhyapradesh_factsheet.pdf.
19. Nebhinani N, Mattoo SK, Wanchu A. HIV Stigma and Specified Correlates in North India. Indian J Psychol Med. 2012;34(4):324-31.

For peer review only - http://bmjopen.bmj.com/site/about/guidelines.xhtml

20. Kumar N, Unnikrishnan B, Thapar R, Mithra P, Kulkarni V, Holla R, et al. Stigmatization and Discrimination toward People Living with HIV/AIDS in a Coastal City of South India. J Int Assoc Provid AIDS Care. 2017;16(3):226-32.

21. Nyblade L, Stangl A, Weiss E, Ashburn K. Combating HIV stigma in health care settings: what works? J Int AIDS Soc. 2009;12:15.

22. Wu S, Li L, Wu Z, Liang LJ, Cao H, Yan Z, et al. A brief HIV stigma reduction intervention for service providers in China. AIDS Patient Care STDS. 2008;22(6):513-20.

23. Shah SM, Heylen E, Srinivasan K, Perumpil S, Ekstrand ML. Reducing HIV stigma among nursing students: a brief intervention. West J Nurs Res. 2014;36(10):1323-37.

24. Pisal H, Sutar S, Sastry J, Kapadia-Kundu N, Joshi A, Joshi M, et al. Nurses' health education program in India increases HIV knowledge and reduces fear. J Assoc Nurses AIDS Care. 2007;18(6):32-43.

25. Nyamathi A, Vatsa M, Khakha DC, McNeese-Smith D, Leake B, Fahey JL. HIV knowledge improvement among nurses in India: using a train-the-trainer program. J Assoc Nurses AIDS Care. 2008;19(6):443-9.

26. Li L, Lin C, Guan J, Wu Z. Implementing a stigma reduction intervention in healthcare settings. J Int AIDS Soc. 2013;16(3 Suppl 2):18710.

27. Aggarwal S, Lee DH, Minteer WB, Fenning RT, Raja SK, Bernstein ME, et al. Another Generation of Stigma? Assessing Healthcare Student Perceptions of HIV-Positive Patients in Mwanza, Tanzania. AIDS Patient Care STDS. 2017;31(2):87-95.

28. Sharma M, Joshi R, Shah H, Macaden R, Lundborg CS. A step-wise approach towards introduction of an alcohol based hand rub, and implementation of front line ownership- using a, rural, tertiary care hospital in central India as a model. BMC Health Serv Res. 2015;15:182.

29. Ganczak M, Barss P, Alfaresi F, Almazrouei S, Muraddad A, Al-Maskari F. Break the silence: HIV/AIDS knowledge, attitudes, and educational needs among Arab university students in United Arab Emirates. J Adolesc Health. 2007;40(6):572 e1-8.

30. Chaudoir SR, Dugan AG, Barr CH. Measuring factors affecting implementation of health innovations: a systematic review of structural, organizational, provider, patient, and innovation level measures. Implement Sci. 2013;8:22.

31. USAID. CAN WE MEASURE HIV/AIDS-RELATED STIGMA AND DISCRIMINATION?
CURRENT KNOWLEDGE ABOUT QUANTIFYING STIGMA IN DEVELOPING COUNTRIES 2006 [Available from: http://www.policyproject.com/pubs/generalreport/Measure%20HIV%20Stigma.pdf.

32. Doda A, Negi G, Gaur DS, Harsh M. Human immunodeficiency virus/acquired immune deficiency syndrome: A survey on the knowledge, attitude, and practice among medical professionals at a tertiary health-care institution in Uttarakhand, India. Asian J Transfus Sci. 2018;12(1):21-6.

33. Mazmanian PE, Davis DA. Continuing medical education and the physician as a learner: guide to the evidence. JAMA. 2002;288(9):1057-60.

34. Davis DA, Thomson MA, Oxman AD, Haynes RB. Changing physician performance. A systematic review of the effect of continuing medical education strategies. JAMA. 1995;274(9):700-5.

35. Li L, Wu Z, Liang LJ, Lin C, Guan J, Jia M, et al. Reducing HIV-related stigma in health care settings: a randomized controlled trial in China. Am J Public Health. 2013;103(2):286-92.

For peer review only

For peer review only - http://bmjopen.bmj.com/site/about/guidelines.xhtml

539 36. Tarja Suominen LL, Dmitry Lioznov, Maya Polukova, Svetlana Nikolaenko, Liudmila Lipiäinen, Maritta Välimäki, Jari Kylmä, Kylmä J. Willingness to take care of HIV positive people: Responses from Russian nursing students. Nordic Journal of Nursing Research. 2015;35(2):71-6.

For peer review only

For peer review only - http://bmjopen.bmj.com/site/about/guidelines.xhtml

Figure 1 Inclusion and exclusion criteria of the study participants

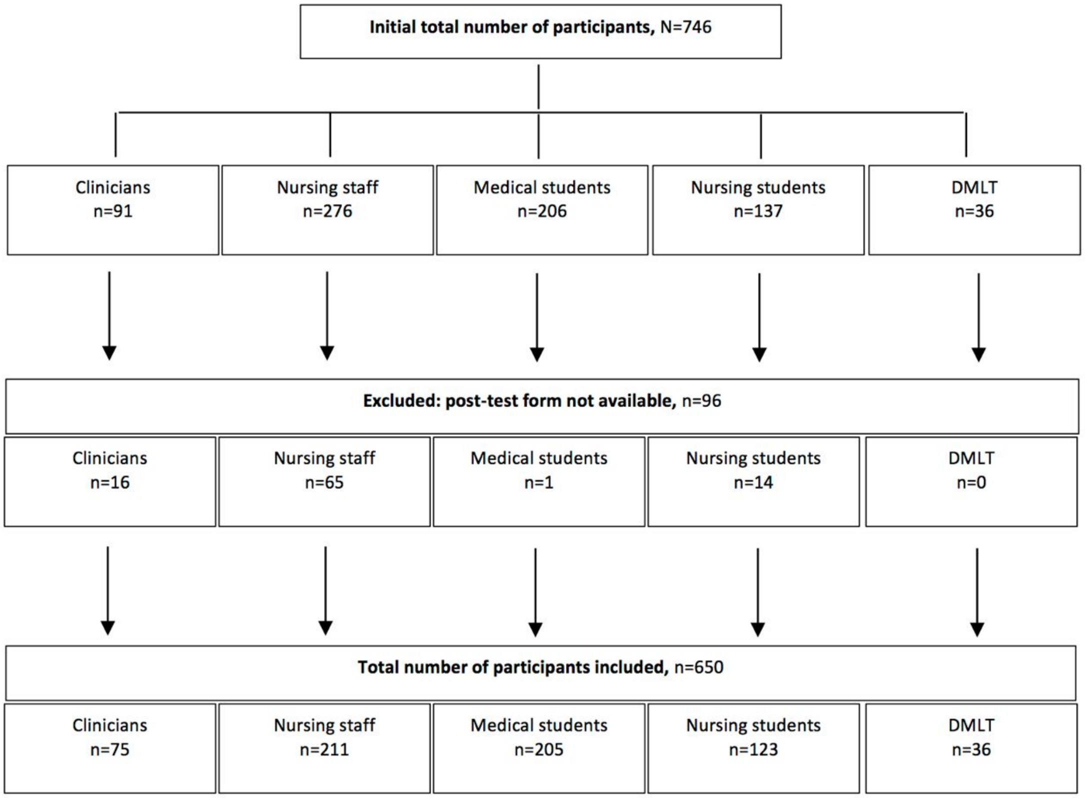

DMLT= Students of Diploma in Medical and Laboratory Technology, Medical students= Students of Bachelor of Medicine, Bachelor of Surgery

Impact of ‘HIV-related stigma-reduction workshops’ on knowledge and attitude of healthcare providers and students in Central India: A pre- and post-test intervention study

Journal:	*BMJ Open*
Manuscript ID	bmjopen-2019-033612.R1
Article Type:	Original research
Date Submitted by the Author:	13-Jan-2020
Complete List of Authors:	Machowska, Anna; Karolinska Institute, Department of Global Public Health<br>Bamboria, Babu Lal; RD Gardi Medical College, Department of Medicine<br>Bercan, Courtney; Karolinska Institutet, Department of Global Public Health<br>Sharma, Megha; Karolinska Institutet, Department of Global Public Health; RD Gardi Medical College, Department of Pharmacology
**Primary Subject Heading**:	Medical education and training
Secondary Subject Heading:	HIV/AIDS, Medical education and training, Health services research, Public health, Occupational and environmental medicine
Keywords:	People living with HIV/AIDS, Stigma and discrimination, Health Care Professionals, HIV-related stigma-reduction workshop, Health & safety < HEALTH SERVICES ADMINISTRATION & MANAGEMENT, MEDICAL EDUCATION & TRAINING

SCHOLARONE™
Manuscripts

I, the Submitting Author has the right to grant and does grant on behalf of all authors of the Work (as defined in the below author licence), an exclusive licence and/or a non-exclusive licence for contributions from authors who are: i) UK Crown employees; ii) where BMJ has agreed a CC-BY licence shall apply, and/or iii) in accordance with the terms applicable for US Federal Government officers or employees acting as part of their official duties; on a worldwide, perpetual, irrevocable, royalty-free basis to BMJ Publishing Group Ltd ("BMJ") its licensees and where the relevant Journal is co-owned by BMJ to the co-owners of the Journal, to publish the Work in this journal and any other BMJ products and to exploit all rights, as set out in our licence.

The Submitting Author accepts and understands that any supply made under these terms is made by BMJ to the Submitting Author unless you are acting as an employee on behalf of your employer or a postgraduate student of an affiliated institution which is paying any applicable article publishing charge ("APC") for Open Access articles. Where the Submitting Author wishes to make the Work available on an Open Access basis (and intends to pay the relevant APC), the terms of reuse of such Open Access shall be governed by a Creative Commons licence – details of these licences and which Creative Commons licence will apply to this Work are set out in our licence referred to above.

Other than as permitted in any relevant BMJ Author's Self Archiving Policies, I confirm this Work has not been accepted for publication elsewhere, is not being considered for publication elsewhere and does not duplicate material already published. I confirm all authors consent to publication of this Work and authorise the granting of this licence.

For peer review only

Impact of 'HIV-related stigma-reduction workshops' on knowledge and attitude of healthcare providers and students in Central India: A pre- and post-test intervention study

Anna Machowska[1#], BL Bamboria[2#], Courtney Bercan[1], Megha Sharma[1,3§]

[1] Department of Global Public Health, Karolinska Institutet, Stockholm, Sweden

[2] Department of Medicine, Ruxmaniben Deepchand Gardi Medical College, Ujjain, India

[3] Department of Pharmacology, Ruxmaniben Deepchand Gardi Medical College, Ujjain, India

[§]Corresponding author:

Dr Megha Sharma

Researcher,

Department of Global Public Health, Karolinska Institutet, Stockholm, Sweden

Associate Professor,

Department of Pharmacology, R.D. Gardi Medical College, Surasa, MP, India

e-mail: megha.sharma@ki.se

[#] These authors have contributed equally to the work

E-mail addresses of authors:

Anna.machowska@ki.se

drblbamboria53@gmail.com

courtney_bercan@hotmail.com

megha.sharma@ki.se, meghasharma27@rediffmail.com

For peer review only

Abstract

Introduction: People living with human immunodeficiency virus (HIV) and acquired immunodeficiency syndrome (AIDS-PLWHA) experience stigma and discrimination throughout their life. The consequences of stigma and discrimination are severe when enacted by healthcare providers (HCPs), and result in a delay in or poor adherence to treatment. Studies have demonstrated the universal presence of stigmatising behaviours among HCPs, yet only a few have presented the effect of interventions on the knowledge and attitude of practising (doctors and nurses) and future HCPs (students).

Objectives: To evaluate knowledge, attitudes and infection risk perceptions related to HIV/AIDS among practising and future HCPs in Central India. In addition, the impact of the 'HIV-related stigma-reduction workshop' using a pre-and post-intervention study design was evaluated.

Settings and participants: The study was conducted in 2016 at two tertiary-care hospitals and among three healthcare colleges in Central India. Overall, 650 HCPs (75 clinicians and 211 nurses) and students (205 medical, 123 nursing and 36 Diploma in Medical and Laboratory Technology [DMLT]) voluntarily participated in the study.

Intervention: The educational intervention comprised of training in epidemiology, the transmission and treatment of the HIV infection, the rights of PLWHA, the duties of HCPs and the use of standard precautions.

Results: At pre-intervention, DMLT students had the lowest and clinicians the highest knowledge scores (24% and 45% respectively). The stigmatised attitude was reflected in all groups, the lowest among clinicians (21%) and the highest among DMLT students (34%). Improvement in the post-

For peer review only

For peer review only - http://bmjopen.bmj.com/site/about/guidelines.xhtml

intervention knowledge scores was the highest in medical students (36%) and the lowest among clinicians (16%). The participants' attitudes improved between 3% and 17% across all groups.

Conclusions: Significant post-intervention improvements were seen in both knowledge and attitudes in all groups. Students had a higher tendency to improve than HCPs. Further long-term studies are needed to evaluate the sustainability of the improvements.

Article Summary

Strengths and limitations of this study

- A context-specific educational intervention was implemented to improve knowledge and attitudes among HCPs and students towards PLWHA.
- The study was conducted at two hospitals and three educational institutes where the participants work and/or study. Thus, we may observe a social desirability bias, resulting in the underestimation of stigma and discrimination among the participants.
- The end goal of any educational intervention is to reduce stigmatising behaviours, but behaviour change is a long-term process that is complex to capture and quantify; thus, attitudes were captured as a proxy.
- The post-intervention questionnaire was completed immediately after the intervention due to feasibility reasons. Thus, we cannot claim the sustainability of the positive effect of the intervention.
- Additional follow-ups would provide stronger evidence of the long-term positive impact of the study on attitudes and knowledge.

Introduction

For peer review only - http://bmjopen.bmj.com/site/about/guidelines.xhtml

The human immunodeficiency virus (HIV) and acquired immunodeficiency syndrome (AIDS) epidemic is a global challenge. At the end of 2017, it was estimated that 36.9 million people were either infected by HIV or living with AIDS (PLWHA) globally. Of these, about 22 million people could access antiretroviral therapy (ART), and 940,000 people had died from HIV-related illnesses (1, 2). The United Nations' 90-90-90 targets launched in 2014 have become a central pillar of global efforts to end the HIV/AIDS epidemic. The aim is to diagnose 90% of all HIV-positive persons, provide ART for 90% of those diagnosed and achieve viral suppression for 90% of those treated by 2020. The program aims to result in 73% of people with HIV achieving viral suppression, a crucial step in ending the AIDS epidemic, by 2030 (3). Stigma, specifically HIV/AIDS-related stigma, is one of the key aspects of the HIV/AIDS epidemic. This stigma can be described as a socially shared perception of the devalued status of PLWHA (4). The presence of stigma and discrimination against PLWHA at various societal levels fuels the HIV epidemic, and the consequences are more severe if the stigma is present among healthcare providers (HCPs).

Healthcare providers are the core and integral part of the healthcare delivery system, and play a pivotal role in creating a positive environment for better health outcomes for PLWHA and preventing viral transmission (5). However, inadequate knowledge about the HIV infection epidemiology among HCPs gives rise to fear and misconceptions about transmission of the infection (6-8). Fear and misconceptions alter attitudes and enhance the development of stigma and discrimination towards PLWHA. Some of the major consequences of this are delays in seeking healthcare, less timely diagnosis, an unwillingness to disclose the HIV status in the community and while seeking healthcare and a delay in or non-compliance to the ART. Not disclosing an HIV status puts society at higher risk of infection transmission, contributes to the increased morbidity and mortality of PLWHA, increases healthcare costs and places a burden on the healthcare sector

For peer review only

For peer review only - http://bmjopen.bmj.com/site/about/guidelines.xhtml

overall (9-11). Therefore, in addition to the diagnosis and treatment, provision of discrimination- and stigma-free healthcare is a prerequisite for achieving the sustainable development target designed to end the epidemic (12).

In 2016, with a prevalence of 2.1 million PLWHA, India was ranked third in the world after South Africa and Nigeria (13, 14). Although the prevalence of PLWHA varies between states in the country, the threat of the infection spreading is high throughout the country due to high levels of inter-state and inter-district migration (15). Global organisations and governments in countries with a high infection risk, including India, have prioritised stigma-reduction through various intervention programs (16). In India, the National AIDS Control Organisation (NACO) advocates the prohibition of social discrimination against PLWHA (14, 16). The organisation has identified seven districts as having high HIV-prevalence; Ujjain is an administrative unit that includes four of these high-risk districts (Neemuech, Mandsour, Ujjain and Ratlam) (17, 18). However, the data available in various parts of the country shows that PLWHA have experienced stigma and discrimination from the community and HCPs (19, 20).

Educational interventions intended to improve knowledge of HIV/AIDS are effective in reducing stigma among HCPs and improving healthcare delivery (12, 21-25). Stigma-reduction intervention studies are, in general, time-consuming and resource-intensive, and most of them have mainly focused on nurses and nursing students, and not on clinicians and healthcare students (10, 22-24). However, medical, laboratory technician and nursing students are future HCPs, which means it is important to develop appropriate interventions to improve their knowledge and reduce their HIV-related stigma while targeting both practising (clinicians and nurses) and future (the students) HCPs (26). However, we know little about HIV/AIDS-related knowledge, attitudes and infection risk perceptions of practising and future HCPs in India.

For peer review only - http://bmjopen.bmj.com/site/about/guidelines.xhtml

Objectives

To evaluate the knowledge, attitudes and infection risk perceptions related to HIV/AIDS among HCPs and healthcare students in the Ujjain district of Central India. Furthermore, to assess and compare the impact of the 'HIV-related stigma-reduction workshop' intervention on the participant groups using a pre-and post-intervention study design.

Methods

This intervention study was conducted between January 2016 and August 2016 at three colleges of healthcare courses and two tertiary-care hospitals in Ujjain district in Madhya Pradesh state in India. An approval was obtained from the ethics committee of R. D. Gardi Medical College, Surasa, Ujjain, India, prior to the study. One of the hospitals is associated with a medical college, a nursing college and a DMLT college and is referred to as the teaching hospital (TH). An ART centre with specialized healthcare facilities for the PLWHAs is located at the TH. Another participating hospital is referred to as the non-teaching hospital (NTH). In this study, the students of Bachelor of Medicine, Bachelor of Surgery (MBBS), are referred to as medical students (27).

Patient and Public Involvement

There was no direct involvement of patients or the public in the present study.

Participants

The participants were practising HCPs, i.e. clinicians and nurses, and future HCPs, i.e. students of medical, nursing and Diploma in Medical and Laboratory Technology (DMLT) courses, enrolled in respective institutes in Ujjain district of Central India.

For peer review only

Sampling strategy

All groups of the HCPs and students were invited to participate in the workshop through various means such as leaflets posted at the notice boards, announcements during lectures or meetings. The leaflets were written in Hindi and English to offer equal chances for all potential participants to learn about and attend the workshop to enhance their HIV/AIDS-related knowledge and ways to stay protected at the workplace. The workshops were conducted at respective colleges and hospitals to provide an equal opportunity to participate.

Inclusion and exclusion criteria

Participation was voluntary, and the participant could leave the session at any time without experiencing any repercussions. The participants who could not attend or complete both the pre- and post-workshop sessions were excluded from the analysis (Figure 1).

Figure 1 Inclusion and exclusion criteria of the study participants Intervention

Structure of the workshops

The intervention was designed as an educational workshop that suited best the local context. All workshops were conducted by the same facilitators: a clinician and an officer-in-charge of the ART centre at the TH (BLB), and the team leader of hospital infection prevention and control team at the TH and the NTH (MS). In total, 13 workshops were conducted. Participants were divided into smaller groups as follows: clinicians (two groups), nurses (five groups), medical students (three groups), nursing students (two groups) and DMLT students (one group) to ensure that they feel comfortable in the learning environment.

For peer review only - http://bmjopen.bmj.com/site/about/guidelines.xhtml

Content of the workshops

The workshop was two hours long and divided into three sessions: the first session consisted of an animated video demonstrating the HIV infection epidemiology, the mechanism of viral transmission, diagnostic testing, treatment options, how the ART drugs work and ethical issues related to PLWHA and ART. This was followed by a second session that involved a PowerPoint presentation focusing on topics such as the rights of PLWHA, the duties of HCPs and predictable consequences due to stigma and discrimination against PLWHA. The third session was more interactive and dynamic. Here, information about universal precautions and infection prevention measures and control was provided, describing the importance of the use of standard precautions, including hand hygiene in the daily routine. It was explained how and when to use gowns, gloves, face shields, masks and goggles, and the availability of post-exposure prophylaxis (PEP) in the study settings and the use of an in-house prepared alcohol-based hand rub (i.e. 'Micro *Kavach*') was discussed (28). This was followed by the demonstration and practice of the WHO-recommended hand hygiene techniques using 'Micro *Kavach*' (28). A period of 15 to 20 minutes was assigned for an open discussion. The participants were asked to complete the questionnaire twice: once before the workshop (pre-intervention) and then after the break (post-intervention).

Data collection tool and analysis

The context-specific questionnaires were developed locally and were used as pre- and post-intervention data collection tools. The questionnaires were pre-tested in each potential participant groups (not included in this study). After receiving feedback, the questionnaires were modified and finalised, and brief educational intervention was designed considering the time constraints among the participants (29). The questionnaires were formatted in both English and Hindi to

For peer review only - http://bmjopen.bmj.com/site/about/guidelines.xhtml

facilitate the understanding of the questions by all participant groups. The questionnaires were divided into the five following sections: (1) participants' demographic information, (2) work experiences, (3) HIV/AIDS-related knowledge, (4) attitudes towards PLWHA, (5) risk perception and willingness to provide care to the PLWHA and patients with Hepatitis B.

The responses related to the knowledge and attitude questions were scored in the following manner: each correct response in the knowledge section received a score of one, and each incorrect response received a score of zero. Scores of each individual were converted to the percentage of correct responses. Like in previous publications in this field; the levels of knowledge were categorised into "low" for the participants who scored ≤50%, "moderate" for those who scored between 51% and 74%, and "high" for those who scored ≥75% (30).

Responses to the questions related to participants' attitude towards PLWHAs were classified into three categories: positive (non-stigmatising), unsure, and negative (stigmatising) (31). A positive response was given a score of one and the responses where the participants were unsure or, where the responses indicated a negative attitude including fear, stigma, or intent to discriminate the PLWHA, were given a score of zero (32).

Scores of each individual were added and converted to the percentage of correct responses. Responses, where the missing data was higher than 50%, were excluded from the analysis. Risk perception questions were categorical and dichotomised according to the outcome of interest for example (a) I am willing or not willing to care for a patient, or (b) I am at high risk or not at high risk of HIV exposure at the workplace.

Statistical analysis

For peer review only - http://bmjopen.bmj.com/site/about/guidelines.xhtml

The data on demographic and basic HIV-related information was presented as mean and standard deviations for continuous variables and as percentages and frequencies for categorical variables. The knowledge, attitudes and work environment variables were presented as median with interquartile range (IQR). The difference between the pre- and post-intervention scores were analysed using a Wilcoxon signed-rank test for continuous variables and McNemar's test for categorical variables. The score difference was calculated by subtracting the pre-intervention (baseline) score from the post-intervention score and represents the percentage unit increase or decrease concerning the baseline score. As these scores were not normally distributed, a Kruskal-Wallis test was conducted to determine a statistically significant difference among the participant groups in pre-intervention and post-intervention scores as well as the difference of scores. Post-hoc Dunn's tests were conducted to evaluate the relationship between the groups. The statistical significance was set at the level of $p<0.05$. The data was entered in Epi info software (version 3.1) and Microsoft Excel and statistical analysis was performed using the Statistical Package for the Social Sciences (SPSS) and STATA 15.0 (Stata Corp. College Station, Texas, USA) software.

Results

Baseline characteristics

In total, 920 people were invited to take part, and 746 (81%) voluntarily participated in the study. Of these, 650 (87%) attended all three sessions of the workshop and completed the questionnaire twice; 56% were students and 44% were practising HCPs. Thus, data from these 650 participants was included in the analysis (Figure 1).

The mean age of the participants was 23 years, the majority were of the Hindu faith and 56% were males (Table 1). The proportion of participants who had received HIV/AIDS-related training

For peer review only - http://bmjopen.bmj.com/site/about/guidelines.xhtml

previously varied from 4% in the clinicians' group to 78% in the DMLT students' group. Only 2% of medical students stated that they had worked with PLWHA before. A high proportion of participants in each group reported experiencing accidental exposure to HIV or PLWHA. A range of 9% to 20% of the participants admitted discriminating against PLWHA in workplaces. Six (3%) of the medical students and up to 11% of DMLT students admitted that they had refused to provide care for PLWHA during their scheduled clinical visits, while between 17% and 28% of participants thought that their colleagues discriminated against PLWHA (Table 1).

Table 1 Baseline demographic information (Part A) and experiences (Part B) of the participants.

	Clinicians (n=75)	Nurses (n=211)	Medical students (n=205)	Nursing students (n=123)	DMLT students (n=36)
**Part A: Baseline demographic information**					
**Age, mean (SD)**	28 (4)	25 (6)	20 (1)	21 (3)	20 (2)
**Sex, n (%)**					
Male	30 (40)	148 (70)	109 (53)	51 (41)	28 (78)
Female	45 (60)	63 (30)	96 (47)	72 (59)	8 (22)
**Married, n (%)**	21 (28)	70 (33)	-	11 (9)	35 (97)
**Religion, n (%)**					
Hindu	62 (83)	192 (91)	183 (89)	120 (98)	31
Muslim	2 (3)	5 (2)	6 (3)	3 (2.4)	4
Christian	2 (3)	10 (5)	1 (0.5)	-	-
Other	9 (12)	4 (2)	15 (7)	-	2
**Have you ever attended any HIV/AIDS related training? Yes, n (%)**	3 (4)	39 (18)	63 (31)	28 (23)	28 (78)
**Part B: Experiences of participants**					
**Have you ever cared for PLWHA before? Yes, n (%)**	37 (49)	121 (57)	4 (2)	88 (72)	18 (50)
If Yes, how many times have you cared for PLWHA?					
Less than 10 times	29 (78)	59 (49)	2 (50)	61 (69)	5 (28)
Less than 50 times	2 (5)	27 (22)	-	14 (20)	8 (44)
More than 100 times	1 (3)	19 (16)	-	-	5 (28)
Cannot recall the numbers /too many	-	16 (13)	2 (50)	13 (15)	-
**§Have you ever had an accidental exposure with body fluid of PLWHA, at work? Yes, n (%)**	§4 (11)	§24 (20)	§3 (75)	§14 (16)	§1 (6)
**Have you ever refused to care for PLWHA? Yes, n (%)**	6 (8)	18 (9)	6 (3)	6 (5)	4 (11)
**Have you discriminated against PLWHA at the workplace? Yes, n (%)**	7 (9)	42 (20)	22 (11)	13 (11)	5 (19)
**Do you think your colleagues discriminate against PLWHA? Yes, n (%)**	21 (28)	45 (21)	51 (25)	28 (23)	6 (17)
**Is your family aware that you ought to care for PLWHA patients as well? Yes, n (%)**	49 (65)	134 (64)	127 (62)	100 (81)	16 (72)

For peer review only - http://bmjopen.bmj.com/site/about/guidelines.xhtml

**Is there any family pressure regarding caring for PLWHAs? Yes, n (%)**	15 (20)	46 (22)	16 (8)	15 (12)	8 (22)
**Do you have any friend or family who is identified as PLWHA? Yes, n (%)**	3 (4)	16 (8)	3 (2)	5 (4)	2 (6)
**There is enough equipment to maintain universal precautions at workplace. Yes, n (%)**	20 (27)	126 (60)	79 (39)	84 (68)	19 (53)
**Have you ever had any accidental exposure to HIV through PLWHA at work? Yes, n (%)**	4 (5)	24 (11)	3 (1)	14 (11)	1 (3)

Yes, n (%): Proportion of the participants who responded "Yes" to the questions asked
DMLT= Students of Diploma in Medical and Laboratory Technology; Medical students= Students of Bachelor of Medicine, Bachelor of Surgery; PLWHA= people living with HIV/AIDS
The boxes where totals do not add up to 100%, the remaining percentage of the participants did not respond to that question. §Proportion calculated from patients who confirmed that they have ever cared for PLWHA.

Impact of the intervention on knowledge scores

The pre-intervention knowledge score, presented as the percentage of correct responses, demonstrated the low overall level of knowledge, with the poorest in DMLT students (22%) and the highest among the clinicians (45%, Table 2). The post-intervention knowledge scores ranged from 48% in the nurses' group to 64% in the medical students' group. The post-intervention knowledge scores were significantly higher than the pre-intervention knowledge scores ($p < 0.001$, Table 2), almost all groups reached a moderate level of knowledge. The difference between the knowledge scores of all study groups was statistically significant ($p<0.001$). The knowledge scores of both medical and nursing students improved more than their respective practising HCPs. A pairwise comparison showed that at the pre-intervention stage, the clinicians had the highest knowledge level of all the groups ($p<0.001$, Table 3). However, at the post-intervention stage, the medical students presented higher knowledge levels than the clinicians, while the knowledge of the nurses was the lowest.

Impact of the intervention on attitude

For peer review only

For peer review only - http://bmjopen.bmj.com/site/about/guidelines.xhtml

Evidence of a stigmatised attitude was observed among all responder groups. The lowest level of stigmatising attitudes at pre-intervention was found in the clinicians' group, where 79% responded to the questions without stigma. On the other hand, in the DMLT students' group, only 66% of responses presented a non-stigmatising attitude (Table 2). At post-intervention, the percentages of non-stigmatising responses were higher, ranging from 79% (lowest) among the nurses to 84% in the medical students' group. The attitude improved significantly across all groups, with a 3% increase in the clinician group and 17% in the DMLT students' group ($p<0.001$). Overall, the intervention resulted in an improvement in stigma levels of up to 21%, as shown in the nurses' group. A pairwise comparison showed that medical students had the greatest improvement in their attitude (Table 4).

Table 2. Pre- and post-intervention scores of knowledge and attitude among the participant groups

**Knowledge**					
	**Clinicians (n=75) % (IQR)**	**Nurses (n=211) % (IQR)**	**Medical students (n=205) % (IQR)**	**Nursing students (n=123) % (IQR)**	**DMLT students (n=36) % (IQR)**
**Percentage of correct responses**					
**Pre-Intervention,** median (IQR)	45 (15)	30 (19)	29 (14)	30 (19)	22 (14)
**Post-Intervention,** median (IQR)	59 (14)	48 (26)	64 (21)	59 (26)	52 (17)
**Difference**	14*	18*	35*	29*	30*
**Attitude**					
**Percentage of responses without stigma**					
**Pre-Intervention,** median (IQR)	79 (18)	74 (15)	74 (18)	76 (12)	66 (35)
**Post-Intervention,** median (IQR)	82 (11)	79 (12)	84 (16)	82 (9)	83 (14)
**Difference**	3*	5*	10*	5*	17*

*Statistically significant differences with significance level $p< 0.05$.
DMLT= Students of Diploma in Medical and Laboratory Technology; IQR= Interquartile range; Medical students= Students of Bachelor of Medicine, Bachelor of Surgery

For peer review only - http://bmjopen.bmj.com/site/about/guidelines.xhtml

Table 3. Pre- and post-intervention pairwise comparison of knowledge between the participant groups

Groups Compared	Pre-Intervention knowledge- Adjusted significance	Post-Intervention knowledge- Adjusted significance	Pre- and Post-Intervention knowledge- Adjusted significance
DMLT students- Nursing students	<0.05*	0.068	1.000
DMLT students - Medical students	<0.01*	<0.001*	0.629
DMLT students -Clinicians	<0.001*	0.116	<0.001*
DMLT students - Nurses	0.059	1.000	0.234
Medical students - Clinicians	<0.001*	0.051	<0.001*
Nursing students - Clinicians	<0.001*	1.000	<0.001*
Nursing students – Medical students	1.000	<0.05*	0.934
Nurses- Nursing students	1.000	<0.001*	<0.001*
Nurses- Clinicians	<0.001*	<0.001*	<0.05*
Nurses– Medical students	1.000	<0.001*	<0.001*

*Statistically significant differences with significance level p< 0.05.
Significance values have been adjusted by the Bonferroni correction for multiple tests. DMLT= Students of Diploma in Medical and Laboratory Technology; Medical students= Students of Bachelor of Medicine, Bachelor of Surgery

Table 4. Pre- and post-intervention pairwise comparison of attitudes between the participant groups

Groups Compared	Pre-Intervention attitude- Adjusted significance	Post-Intervention attitude- Adjusted significance	Pre- and Post-Intervention attitude- Adjusted significance
DMLT students - Nursing students	0.184	1.000	0.147
DMLT students - Medical students	0.479	0.614	1.000
DMLT students -Clinicians	<0.01*	1.000	0.134
DMLT students - Nurses	0.627	1.000	0.439
Medical students - Clinicians	<0.05*	1.000	<0.01*
Nursing students - Clinicians	0.252	1.000	1.000
Nursing students – Medical students	1.000	0.173	<0.01*
Nurses- Nursing students	1.000	0.614	1.000
Nurses- Clinicians	<0.05*	0.092	1.000
Nurses– Medical students	1.000	<0.001*	<0.05*

*Statistically significant differences with significance level p< 0.05. Significance values have been adjusted by the Bonferroni correction for multiple tests.
DMLT= Students of Diploma in Medical and Laboratory Technology; Medical students= Students of Bachelor of Medicine, Bachelor of Surgery

For peer review only - http://bmjopen.bmj.com/site/about/guidelines.xhtml

Table 5. Risk perception reported by the participants in the pre- and post-intervention questionnaires.

Questions	Time points	Nurses (n=211) n (%)	Clinicians (n=75) n (%)	Nursing students (n=123) n (%)	Medical students (n=205) n (%)	DMLT students (n=36) n (%)
Do you think you are at high risk for HIV/AIDS at work? Yes, n (%)	Pre-intervention	71 (34)	50 (67)	39 (32)	61 (30)	22 (61)
	Post-intervention	81 (38)	51 (68)	86 (70)*	82 (40)*	17(47)
Are you willing to care for- a) Patients with hepatitis B (Yes), n (%)	Pre-intervention	174 (83)	64 (85)	112 (91)	155 (76)	28 (78)
	Post-intervention	192 (91)*	65(87)	118 (96)	196 (96)*	32 (89)
b) PLWHA§ (Yes), n (%)	Pre-intervention	151 (72)	65 (87)	114 (93)	179 (87)	34 (94)
	Post-intervention	175 (83)*	66 (88)	110 (89)	183 (89)	30 (83)

Yes, n (%): Proportion of the participants who responded "Yes" to the questions asked
*Statistically significant differences with significance level $p < 0.05$. DMLT= Students of Diploma in Medical and Laboratory Technology; Medical students= Students of Bachelor of Medicine, Bachelor of Surgery; PLWHA= People living with HIV/AIDS. [§]The question was also classified as an attitude question.

Impact of the intervention on infection risk perception

Thirty per cent of the medical students and 67% of the clinicians perceived themselves to be at high risk of HIV infection in their workplace at the pre-intervention stage. The willingness to care for PLWHA was relatively high at the pre-intervention stage, ranging from 72% among the nurses to 94% among the DMLT student group. The proportion of the participants who were willing to care for people living with Hepatitis B was similar to those willing to care for PLWHA.

After the workshop, the medical and nursing students reported a significant increase in their awareness of the risk of HIV infection compared to their statements at pre-intervention. There was a significant increase in the post-intervention willingness to care for PLWHA in the nursing group, and a non-significant decrease in the DMLT and nursing students' group (Table 5).

For peer review only - http://bmjopen.bmj.com/site/about/guidelines.xhtml

Discussion

The present study evaluated the effect of 'HIV-related stigma-reduction workshops' and showed that there was a significant improvement in HIV/AIDS-related knowledge in all groups who participated in the intervention, irrespective of their inadequate baseline knowledge. Our study highlighted the fact that stigmatising attitudes were widespread among practising HCPs and students of various healthcare courses. However, stigmatising attitudes and discrimination that led to an unwillingness to provide medical care to PLWHA were significantly reduced among the participants after the intervention. The student groups showed more receptiveness to improve their knowledge and attitudes towards PLWHA than the practising HCPs.

Knowledge

Low knowledge levels can perpetuate myths and misunderstandings that extend to baseline beliefs and stigmatising attitudes. The present study showed that while the baseline HIV knowledge of both the practicing HCPs and the healthcare students was inadequate, there was a significant knowledge improvement in all participant groups after the intervention. These findings are comparable with results from a cross-sectional survey conducted among practicing HCPs and healthcare students in Uttarakhand, India (32). That study also found low levels of HIV-related knowledge among HCPs and highlighted the need for improved HIV-specific education. Our study echoed their finding that clinicians had more knowledge than the other groups. This is understandable, as physicians are likely to receive more HIV-specific training than other types of HCPs (33).

In our study, the overall post-intervention knowledge scores significantly increased in all groups, and particularly so in the students' groups. This was similar to the effect of educational

For peer review only - http://bmjopen.bmj.com/site/about/guidelines.xhtml

interventions on HIV knowledge in two single-centre studies involving nursing students in Bengaluru and nurses in Delhi, India (22, 24). In Bengaluru, the intervention was divided into two one-hour sessions, while in Delhi it involved a two-day training program.

We hypothesised that there would be a greater post-intervention increase in knowledge among the clinicians' group than the other groups. However, the students' groups showed significant knowledge gains and the clinicians showed the lowest increase. The theories suggest that knowledge is associated with baseline beliefs and attitudes, and depends on the readiness of the learners and their willingness to integrate the change into their behaviour (34, 35). As per the educational theory, the recognition of the gap in knowledge and skills, or the need for behaviour change, is essential for making the desired change (34, 35). The student groups, being in the learning phase of life, may have identified their gaps in knowledge, thus making them more receptive to the training than the other groups (34, 35), while the clinicians had adequate knowledge at pre-intervention and thus had less scope to improve their knowledge at the post-intervention stage.

Attitude

Stigmatising attitudes towards PLWHA can feed into a lack of desire or willingness to learn more about the disease. The present study confirmed our hypothesis that stigmatising attitudes are widespread among practicing HCPs and healthcare students in the region. Our pre-intervention stigma levels were higher in healthcare students and lower in practicing HCPs, which is consistent with a cross-sectional study of HCPs in Uttarakhand, India (33). Professional experience with PLWHA is a predictor of more positive attitudes towards them (14, 27). The higher levels of

For peer review only

stigmatising attitudes found among participants (students) in our study could be due to incomplete or inadequate knowledge, and relatively less experience of working with PLWHA.

Studies show that working on empathy-building techniques and including PLWHA testimonies in interventions can improve outcomes for HCPs and contribute to decreasing the stigma and discrimination towards PLWHA (36, 37). Recent studies demonstrate that small efforts such as support in the workplace, HCP-friendly policies and displaying stigma-reduction messages can change HCPs' attitudes towards PLWHA.

Similar to the knowledge scores, the student groups showed larger improvements in their stigmatising attitudes than the working professionals. We must acknowledge, however, that although improvements in knowledge were seen in all groups, increased knowledge is not typically enough to affect changes in a person's attitudes and the baseline beliefs that contribute to stigma and discrimination. These changes are the result of a complex interplay between knowledge, baseline beliefs and attitudes that affects the readiness of the learners and their willingness to integrate the change into their behaviour (10, 34, 35).

Risk perception

Another highlight of our study was the willingness to care for PLWHA shown by the majority of HCPs and students at the pre-intervention stage. Our findings are in line with a study conducted in India by Kermode et al. that showed a relatively high willingness of HCPs to care for PLWHA despite the presence of stigmatising attitudes (7). However, there are few studies that assess risk perception prior to and after a stigma-reduction intervention.

For peer review only - http://bmjopen.bmj.com/site/about/guidelines.xhtml

Medical and nursing students significantly improved their perception of HIV/AIDS risk in the post-intervention test. The same receptiveness to learning that was shown by the student groups in our study is likely to be the reason their knowledge in this area also improved. It must be said, however, that educational interventions may have a limited effect on risk perception if appropriate personal protective materials are not available in the workplace. This points to the necessity to create structural and policy changes that contribute to a safer work environment in conjunction with educational interventions in order to both reduce perceived and actual workplace risk (6, 7). Further studies are needed to determine the factors affecting HCPs' willingness to care for PLWHA.

Our study is based on the call for new research that promotes the development of knowledge-building and stigma-reduction interventions to target the broad range of HCPs that PLWHA will encounter in their healthcare journeys (38, 39). The scaling-up of HIV stigma-reduction efforts lacks focus throughout the world, and especially in countries with a high HIV prevalence like India. The major challenge is the implementation feasibility of the interventions, mainly due to the time commitment and resources required.

The number and duration of educational sessions does not necessarily correspond to greater increases in knowledge, and shorter interventions may suffice to bring participants to an adequate level of knowledge (38, 39). Researchers have used various time lengths to assess their intervention designs, and thus it is not appropriate to conclude there is a relationship between the length and number of workshops and the level of change (22, 24, 36, 40). However, it seems that even a short workshop can produce positive results, as seen by the progress of the students' groups in our study.

For peer review only - http://bmjopen.bmj.com/site/about/guidelines.xhtml

Strengths and limitations

To the best of our knowledge, this is the first study that presents knowledge and stigma in terms of attitudes towards PLWHA among various groups of practising and future HCPs. Context-specific low-cost educational intervention was feasible and appreciated by most of the HCPs during both the pilot testing and the real study. Our intervention is brief and requires relatively few resources and little time, which is important in settings where HCPs work. These features increase the generalisability and feasibility of adopting the intervention design and applying it in other similar settings where HCPs are burdened by a heavy workload.

This study shows that an educational and skills-based intervention, even if only for two hours, can be effective in increasing knowledge and reducing stigmatised attitudes. Therefore, recurrent training and educational workshops might have a sustainable effect of improved knowledge and reduced stigmatised attitudes and behaviour towards PLWHA. Finally, the present study fills a knowledge gap by providing a better understanding of the knowledge, attitudes and risk perceptions of various HCPs and healthcare students in India and globally.

Like any study, this methodology must be seen in the context of its limitations. The major consideration of our study is that the post-intervention questionnaire was completed after a short break following the intervention. Thus, we could not validate whether the reduction in stigmatising beliefs was sustained over time. The end goal of the intervention was to reduce stigmatised behaviour. However, behaviour change is a long-term process and is complex to capture and quantify; thus, we were restricted to capturing attitudes as a proxy. Although the present one-time post-intervention data collection design presents a strong association between the intervention and the results, additional follow-ups, for example at four weeks, six months and one year, would

For peer review only

provide stronger evidence for the positive impact of the study on attitudes and knowledge than the current design.

Given that the study was conducted in workplaces, the presence of social desirability bias in participants' stigma-related responses cannot be ruled out. Therefore, it is possible that the rate of stigma presented in the study is underestimated. The voluntary participation, with an emphasis on increasing knowledge, and the anonymous questionnaire completion, might have motivated HCPs to participate in the study.

Conclusions

Combating stigma is a key priority in achieving success in epidemic control and providing quality care to PLWHA. The findings from this study show that the HIV-related stigma-reduction workshops that are brief, feasible and relatively low-resource interventions, can successfully increase knowledge and reduce stigma in both HCPs and students. Considering the generalisability and feasibility of the intervention design, repetitive contextualised workshops with extended follow-ups are recommended to evaluate their impact and assure sustainable effect. We propose incorporating of more practical and integrated educational training in the teaching curricula of all healthcare courses. This will benefit the HCPs, PLWHAs and the community. Moreover, in future studies, we recommend the inclusion of a qualitative component and behavioural assessment to understand participants' attitudes and willingness to change stigmatising behaviour towards PLWHA.

Acknowledgements

For peer review only

For peer review only - http://bmjopen.bmj.com/site/about/guidelines.xhtml

We thank all study participants, the management and staff of the hospitals and colleges for facilitating and supporting the study. We are thankful to Mr Amit Pawar for assisting in data collection and data management.

<u>Footnotes</u>

Author contributions

MS and BLB perceived the study. MS and BLB designed the questionnaire and the content of the interventions. MS and BLB conducted the training sessions and collected the pre-and post-intervention data. AM, CB and MS performed the analysis. AM and MS drafted the article. AM, MS, BLB and CB gave critical comments to modify the manuscript. All authors gave final approval to the present version for publication.

Funding

This work was supported by Swedish Research Council grant number 2017-01327.

Competing interests

Authors have no conflicts of interests to declare.

Ethical considerations

Ethical approval was granted by the ethics committee of R. D. Gardi Medical College, Surasa, Ujjain, India with letter numbers 311/17-07-2013 and 249/23-01-2013. Verbal and written consents were obtained from each participant before the study. Participants were assured by the

For peer review only

For peer review only - http://bmjopen.bmj.com/site/about/guidelines.xhtml

researchers for full confidentiality. Participants were encouraged to respond to the questionnaires fearlessly and were ensured for no repercussions based on the responses.

A data sharing statement

All data generated during the study are included in the article. The data are not publicly available at present because it contains information that could compromise the confidentiality or consent of the study participants.

Patient consent for publication

Not required.

For peer review only

References

1. The WHO. Sustainable Development Goals 2015 [Available from: http://www.un.org/sustainabledevelopment/sustainable-development-goals/.
2. The WHO. Global Health Observatory (GHO) data: HIV/AIDS 2019 [Available from: https://www.who.int/gho/hiv/en/.
3. UNAIDS. 90–90–90—An ambitious treatment target to help end the AIDS epidemic 2017 [Available from: https://www.unaids.org/en/resources/documents/2017/90-90-90.
4. HIV/AIDS JUNPo. Stigma and discrimination 2003 [Available from: http://data.unaids.org/Publications/fact-sheets03/fs_stigma_discrimination_en.pdf.
5. Chambers LA, Rueda S, Baker DN, Wilson MG, Deutsch R, Raeifar E, et al. Stigma, HIV and health: a qualitative synthesis. BMC Public Health. 2015;15:848.
6. Datta S, Bhattacherjee S, Sherpa PL, Banik S. Perceived HIV Related Stigma among Patients Attending ART Center of a Tertiary Care Center in Rural West Bengal, India. J Clin Diagn Res. 2016;10(10):VC09-VC12.
7. Kermode M, Holmes W, Langkham B, Thomas MS, Gifford S. HIV-related knowledge, attitudes and risk perception amongst nurses, doctors and other healthcare workers in rural India. Indian J Med Res. 2005;122(3):258-64.
8. Vorasane S, Jimba M, Kikuchi K, Yasuoka J, Nanishi K, Durham J, et al. An investigation of stigmatizing attitudes towards people living with HIV/AIDS by doctors and nurses in Vientiane, Lao PDR. BMC Health Serv Res. 2017;17(1):125.
9. Steward WT, Bharat S, Ramakrishna J, Heylen E, Ekstrand ML. Stigma is associated with delays in seeking care among HIV-infected people in India. J Int Assoc Provid AIDS Care. 2013;12(2):103-9.
10. Ekstrand ML, Ramakrishna J, Bharat S, Heylen E. Prevalence and drivers of HIV stigma among health providers in urban India: implications for interventions. J Int AIDS Soc. 2013;16(3 Suppl 2):18717.
11. Katz IT, Ryu AE, Onuegbu AG, Psaros C, Weiser SD, Bangsberg DR, et al. Impact of HIV-related stigma on treatment adherence: systematic review and meta-synthesis. J Int AIDS Soc. 2013;16(3 Suppl 2):18640.
12. Wu S, Li L, Wu Z, Liang LJ, Cao H, Yan Z, et al. A brief HIV stigma reduction intervention for service providers in China. AIDS Patient Care STDS. 2008;22(6):513-20.
13. UNAIDS data. 2017 [Available from: http://www.unaids.org/sites/default/files/media_asset/20170720_Data_book_2017_en.pdf.
14. National Aids Control Organization. 2016-2017 [Available from: http://naco.gov.in/sites/default/files/NACO%20ANNUAL%20REPORT%202016-17.pdf.
15. Madhya Pradesh State AIDS Control Society. [Available from: http://www.mpsacsb.org/MPSACS-introduction.php.
16. Bagcchi S. India passes law prohibiting discrimination against people with HIV/AIDS. BMJ. 2017;357:j1973.
17. Office of the Registrar General & Census Commissioner. Census of India 2011 [Available from: http://censusindia.gov.in/2011-prov-results/data_files/mp/01Content.pdf.
18. Economic and Human Development Indicators 2011 [Available from: http://www.undp.org/content/dam/india/docs/madhyapradesh_factsheet.pdf.

For peer review only - http://bmjopen.bmj.com/site/about/guidelines.xhtml

19. Nebhinani N, Mattoo SK, Wanchu A. HIV Stigma and Specified Correlates in North India. Indian J Psychol Med. 2012;34(4):324-31.

20. Kumar N, Unnikrishnan B, Thapar R, Mithra P, Kulkarni V, Holla R, et al. Stigmatization and Discrimination toward People Living with HIV/AIDS in a Coastal City of South India. J Int Assoc Provid AIDS Care. 2017;16(3):226-32.

21. Nyblade L, Stangl A, Weiss E, Ashburn K. Combating HIV stigma in health care settings: what works? J Int AIDS Soc. 2009;12:15.

22. Shah SM, Heylen E, Srinivasan K, Perumpil S, Ekstrand ML. Reducing HIV stigma among nursing students: a brief intervention. West J Nurs Res. 2014;36(10):1323-37.

23. Pisal H, Sutar S, Sastry J, Kapadia-Kundu N, Joshi A, Joshi M, et al. Nurses' health education program in India increases HIV knowledge and reduces fear. J Assoc Nurses AIDS Care. 2007;18(6):32-43.

24. Nyamathi A, Vatsa M, Khakha DC, McNeese-Smith D, Leake B, Fahey JL. HIV knowledge improvement among nurses in India: using a train-the-trainer program. J Assoc Nurses AIDS Care. 2008;19(6):443-9.

25. Li L, Lin C, Guan J, Wu Z. Implementing a stigma reduction intervention in healthcare settings. J Int AIDS Soc. 2013;16(3 Suppl 2):18710.

26. Aggarwal S, Lee DH, Minteer WB, Fenning RT, Raja SK, Bernstein ME, et al. Another Generation of Stigma? Assessing Healthcare Student Perceptions of HIV-Positive Patients in Mwanza, Tanzania. AIDS Patient Care STDS. 2017;31(2):87-95.

27. Landstedt K, Sharma A, Johansson F, Stalsby Lundborg C, Sharma M. Antibiotic prescriptions for inpatients having non-bacterial diagnosis at medicine departments of two private sector hospitals in Madhya Pradesh, India: a cross-sectional study. BMJ Open. 2017;7(4):e012974.

28. Sharma M, Joshi R, Shah H, Macaden R, Lundborg CS. A step-wise approach towards introduction of an alcohol based hand rub, and implementation of front line ownership- using a, rural, tertiary care hospital in central India as a model. BMC Health Serv Res. 2015;15:182.

29. Ruotsalainen JH, Verbeek JH, Marine A, Serra C. Preventing occupational stress in healthcare workers. Cochrane Database Syst Rev. 2015(4):CD002892.

30. Ganczak M, Barss P, Alfaresi F, Almazrouei S, Muraddad A, Al-Maskari F. Break the silence: HIV/AIDS knowledge, attitudes, and educational needs among Arab university students in United Arab Emirates. J Adolesc Health. 2007;40(6):572 e1-8.

31. Chaudoir SR, Dugan AG, Barr CH. Measuring factors affecting implementation of health innovations: a systematic review of structural, organizational, provider, patient, and innovation level measures. Implement Sci. 2013;8:22.

32. USAID. Can we measure HIV/AIDS-related stigma and discrimination? Current knowledge about quantifying stigma in developing countries 2006 [Available from: http://www.policyproject.com/pubs/generalreport/Measure%20HIV%20Stigma.pdf.

33. Doda A, Negi G, Gaur DS, Harsh M. Human immunodeficiency virus/acquired immune deficiency syndrome: A survey on the knowledge, attitude, and practice among medical professionals at a tertiary health-care institution in Uttarakhand, India. Asian J Transfus Sci. 2018;12(1):21-6.

34. Mazmanian PE, Davis DA. Continuing medical education and the physician as a learner: guide to the evidence. JAMA. 2002;288(9):1057-60.

For peer review only - http://bmjopen.bmj.com/site/about/guidelines.xhtml

35. Davis DA, Thomson MA, Oxman AD, Haynes RB. Changing physician performance. A systematic review of the effect of continuing medical education strategies. JAMA. 1995;274(9):700-5.
36. Batey DS, Whitfield S, Mulla M, Stringer KL, Durojaiye M, McCormick L, et al. Adaptation and Implementation of an Intervention to Reduce HIV-Related Stigma Among Healthcare Workers in the United States: Piloting of the FRESH Workshop. AIDS Patient Care STDS. 2016;30(11):519-27.
37. Pulerwitz J, Oanh KT, Akinwolemiwa D, Ashburn K, Nyblade L. Improving hospital-based quality of care by reducing HIV-related stigma: evaluation results from Vietnam. AIDS Behav. 2015;19(2):246-56.
38. Andersson GZ, Reinius M, Eriksson LE, Svedhem V, Esfahani FM, Deuba K, et al. Stigma reduction interventions in people living with HIV to improve health-related quality of life. Lancet HIV. 2019.
39. Safreed-Harmon K, Anderson J, Azzopardi-Muscat N, Behrens GMN, d'Arminio Monforte A, Davidovich U, et al. Reorienting health systems to care for people with HIV beyond viral suppression. Lancet HIV. 2019;6(12):e869-e77.
40. Srithanaviboonchai K, Stockton M, Pudpong N, Chariyalertsak S, Prakongsai P, Chariyalertsak C, et al. Building the evidence base for stigma and discrimination-reduction programming in Thailand: development of tools to measure healthcare stigma and discrimination. BMC Public Health. 2017;17(1):245.

For peer review only

For peer review only - http://bmjopen.bmj.com/site/about/guidelines.xhtml

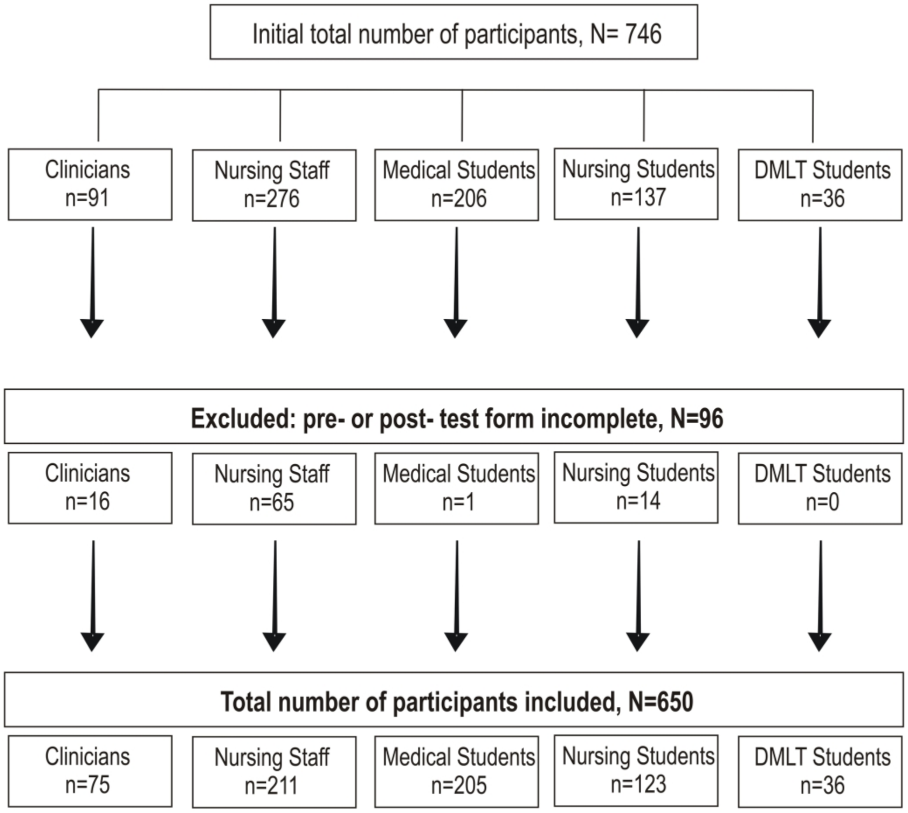

Figure 1 Inclusion and exclusion criteria of the study participants Intervention

125x111mm (600 x 600 DPI)
